# Antenna Deployment for the Localization of Partial Discharges in Open-Air Substations

**DOI:** 10.3390/s16040541

**Published:** 2016-04-15

**Authors:** Guillermo Robles, José Manuel Fresno, Matilde Sánchez-Fernández, Juan Manuel Martínez-Tarifa

**Affiliations:** 1 Department of Electrical Engineering, Universidad Carlos III de Madrid, Avda, Universidad, 30, Leganés, Madrid 28911, Spain; jfresno@ing.uc3m.es (J.M.F.); jmmtarif@ing.uc3m.es (J.M.M.-T.); 2 Department of Signal Theory and Communications, Universidad Carlos III de Madrid, Avda, Universidad, 30, Leganés, Madrid 28911, Spain; mati@tsc.uc3m.es

**Keywords:** antennas, radio-frequency localization, partial discharges, particle swarm optimization

## Abstract

Partial discharges are ionization processes inside or on the surface of dielectrics that can unveil insulation problems in electrical equipment. The charge accumulated is released under certain environmental and voltage conditions attacking the insulation both physically and chemically. The final consequence of a continuous occurrence of these events is the breakdown of the dielectric. The electron avalanche provokes a derivative of the electric field with respect to time, creating an electromagnetic impulse that can be detected with antennas. The localization of the source helps in the identification of the piece of equipment that has to be decommissioned. This can be done by deploying antennas and calculating the time difference of arrival (TDOA) of the electromagnetic pulses. However, small errors in this parameter can lead to great displacements of the calculated position of the source. Usually, four antennas are used to find the source but the array geometry has to be correctly deployed to have minimal errors in the localization. This paper demonstrates, by an analysis based on simulation and also experimentally, that the most common layouts are not always the best options and proposes a simple antenna layout to reduce the systematic error in the TDOA calculation due to the positions of the antennas in the array.

## 1. Introduction

The maintenance of high-voltage (HV) equipment, namely electrical machines and power cables, is essential for the reliability of the power grid. Specifically, power transformers and other HV equipment in substations must be in good condition to ensure that the flow of electrical power is not interrupted. The electrical insulation in these assets becomes the weakest factor due to electrical, thermal and environmental stresses that, combined, can lead to unexpected failures [1,2]. These ageing agents can cause partial discharges (PD). These are low-energy ionizations that are not harmful in short periods of time, but that have certain degradation capability by themselves [3]. Their detection can be used for the diagnosis of this electrical equipment [4], and helps in early warning systems to detect which piece of high voltage equipment is prone to failure. These types of events emit energy in radio-frequency bands so they can be detected with antennas [5,6]. Moreover, if a set of antennas is strategically placed nearby the asset, the PD source could be located accurately.

There are different location techniques in the literature [7,8] that could be classified as triangulation, received signal strength (RSS) or proximity. All three of them have advantages and drawbacks and not all of them are suitable for all scenarios. Proximity is the least complex in terms of processing but it also has the worst performance in terms of accuracy since it only provides information on which is the closest antenna to the device that is to be located and therefore places the device in this antenna coverage area. RSS needs to have information on the power of the signal transmitted so it is not an alternative that is suitable for scenarios where this characteristic of the signal is unknown. Finally, triangulation is based on geometry. It needs at least three reference antennas and works either with the time of arrival (TOA) or with the time difference of arrival (TDOA) of the signals to the different antennas. For the TOA, it is necessary that the antennas and the source generating the location signal have the same time reference, which again is unfeasible in our scenario where the occurrence of the partial discharges is random. The TDOA only needs the time difference between the received signals in each pair of antennas. Given all this, in a scenario where the position of equipment that randomly emits a PD is required, TDOA arises as the most adequate technique.

There are many references devoted to the localization of partial discharges inside transformers and GIS substations based on TDOA [9,10,11,12]. Placing the antennas in these cases is straightforward and they simply have to be deployed around the source to have an accurate localization. The problem arises when the position of the source is not confined in a known piece of equipment and, then, cannot be surrounded by the antennas. In this case, which is especially important when localizing PD sources in open-air substations, there is a extremely large uncertainty in the position of the source due to errors in the TDOA. The reason for this mismatch on performance depending on the position of the PD source with respect to the polygon defined by the antennas is intrinsic to the solution that provides the TDOA. This solution is obtained by calculating the intersections of hyperbolas with foci in every pair of antennas. The lines defined by the hyperbolas are more concentrated inside the polygon than outside so the set of possible solutions shows less dispersion when the source is surrounded by the antennas. There is an interesting paper by Moore *et al.* that studies the location performance of two configurations forming a square and a star determining that the first one is the best option [13]. There is also a mathematical study in [14] that concludes with some evident hints about how to place the antennas and test them in a square layout. Again, the square configuration is used in [15,16,17]. The authors in [16] further included a trapezoidal layout and tested the antenna deployments in a 400 kV substation obtaining an experimental measure of the statistical error. However, the sources were included inside the polygon of the antenna layout so their analysis is different from what it is proposed in this paper. In our study we propose a trapezoidal layout forcing the PD source to be outside the polygon defined by the array. We show that, both through a realistic modelling of different antenna deployments that takes into account errors in the measurement of the TDOA and by experimental measurements, a trapezoidal array improves the performance of a squared deployment, having better accuracy and less dispersion than other configurations.

## 2. Finding the Radio-Frequency Source

This section defines the mathematical frame and geometrical relations that establish the base of the simulation. Partial discharges are pulsed events that emit an electromagnetic radiation. The speed of propagation, *c*, the distance, $D_{i}$ and the time $t_{i}$ that takes a pulse placed in $P_{s}=\left(x_{s},y_{s},z_{s}\right)$ to propagate in free space to the antenna *i* in $P_{i}=\left(x_{i},y_{i},z_{i}\right)$ is related by:

(1) $$D_{i}=c\cdot t_{i}=\sqrt{{\left(x_{i}-x_{s}\right)}^{2}+{\left(y_{i}-y_{s}\right)}^{2}+{\left(z_{i}-z_{s}\right)}^{2}}=∥P_{i}-P_{s}∥$$

Unfortunately, as it was previously mentioned, the onset time of the partial discharge is not known so the absolute time of arrival cannot be used and it is necessary to measure the time difference of arrival to every pair of antennas to find the source position. The equation is then changed to:

(2) $$D_{ij}=c\left(t_{i}-t_{j}\right)=∥P_{i}-P_{s}∥-∥P_{j}-P_{s}∥$$

The unknowns are the three components of the location of the source so it is necessary to have at least three equations and solve the system to find the position. Considering four antennas, we could choose to extend Equation (2) to:

(3) $$\begin{array}{ccc} D_{12} & = & c\left(t_{1}-t_{2}\right)=∥P_{1}-P_{s}∥-∥P_{2}-P_{s}∥\\ D_{13} & = & c\left(t_{1}-t_{3}\right)=∥P_{1}-P_{s}∥-∥P_{3}-P_{s}∥\\ D_{14} & = & c\left(t_{1}-t_{4}\right)=∥P_{1}-P_{s}∥-∥P_{4}-P_{s}∥ \end{array}$$

which constitutes a system of non-linear equations.

The exact determination of the TDOA, $t_{i}-t_{j}$, is key to have an accurate position of the source and small variations in this parameter can induce a large misplacement [13].

There are multiple factors that can lead to uncertainties or even errors in the TDOA. For example, the uncertainties produced by the intrinsically digital nature of the sampled signal and the system sampling rate, lead to a region in space inside which it would be impossible to locate the PD; while errors in the TDOA lead to wrong positioning. These factors have different origins that are classified in this paper into three categories: due to the nature of the signal, due to the position of the antennas and due to the measuring procedure.

### 2.1. Uncertainties and Errors in the TDOA due to the Nature of the Signal

To obtain the TDOA, $t_{i}-t_{j}$, it is necessary to extract information from the signals received in each antenna, *i.e.*, we should be able, in the case of a PD, to identify the onset of the pulse. There are several aspects of the nature of the PD waveform that may affect this identification negatively, such as the rise slope of the PD wavefront, the presence of noise and interferences in the propagation bandwidth and the propagation through multipaths.

In the absence of noise or any other perturbations, the sharper the rise slope a PD presents, the more precise it will be to determine the onset of the pulse. The waveform of a PD depends on the type of insulation where it happens. The signal emitted by corona or surface discharges, where the insulation interfaces are air or air-dielectric, will be different from PD discharging inside the solid or liquid insulation because the wave has to pass through the dielectric before traveling through air to the detectors. Also, weather conditions such as temperature, humidity and pressure affect to partial discharge inception voltages and hence the pulsed signal emitted during the discharge. Additionally, PD are intrinsically random signals, and therefore they need to be modeled as stochastic processes. The randomness comes from the fact that the charge involved in the discharge and the path followed by the electron avalanche change in every event [18]. For this reason, this phenomenon cannot be analysed using only one PD event and the signal received in the antennas will not always have the same rise time or wavefront type.

In most propagation scenarios, and specifically for measurements in open-air substations, the partial discharge pulse is contaminated by interferences in all radio-frequency spectrum such as FM radio, TV broadcasting, mobile communications, WiFi, lighting, commutations, sparking and arcing. All these noise sources can ultimately degrade the signal-to-noise ratio (SNR) hindering the process of finding the onset of the pulse. These uncertainties in picking the initial time will add an error to the measurement of the TDOA. There are well-known algorithms to find the initial time of a pulse such as cross correlation with or without preprocessor, first introduced in [19]; the detection of steep changes in the cumulative energy of the time signal [9]; or the modification of this method including a negative trend [20,21]; the Akaike information criterion (AIC) based on autoregressive processes; and, energy-based methods using high order statistics such as kurtosis and skewness [22]. Some benchmarks can be found in [21,23].

Finally, the last significant source of uncertainty and errors is the lack of line-of-sight or the presence of obstacles that produce reflections in the signal. These reflections jeopardize the measurement of the time of arrival in Equation (1) given the fact that this formula assumes that the propagation is in free space. Then, if there are metallic structures between the source and the antenna, the signal received would be distorted, the wavefront rise-time would not be as fast as it should be and the effect of multipath reflections could hinder the obtention of the TDOA.

### 2.2. Errors in the TDOA due to the Geometry and Radiation Characteristics of the Antennas

There are several parameters of the antenna array that have a clear influence in the estimation of the TDOA and in the process of calculating the location of the source: the position of each antenna, the radiation pattern and the bandwidth.

Setting a reference system to place the antennas is essential, since the exact situation of each antenna is needed to further extract the position of the source from the TDOA. Then, the location of the antennas has to be measured carefully because small errors in the order of a few centimeters will be translated to a large dispersion in the results. Eventually, choosing an appropriate layout can reduce the uncertainty in the localization of the source as demonstrated in the next sections. Moreover, the total size of the antenna array determines where the far field region is placed so that the antennas can be dimensionally negligible to place them correctly in a single point in space.

The two remaining parameters may modify the PD waveform, and, consequently, lead to errors in the measurement of the TDOA, as also explained in Section 2.1. The radiation pattern of the antenna and the antenna bandwidth play an important role in the localization process, since they can create difficulties in the identification of the pulse onset. If the source is located below the ground plane of the antenna, the rise time of the received signal could be slower, so all TDOA involving that antenna would induce a measurement error. Furthermore, the antenna acts as a filter modifying the received waveform according to the antenna frequency response. In this scenario, the antenna bandwidth should be broader than the PD bandwidth, or, at least, high enough so as to not affect the raising slope of the pulse [24].

### 2.3. Uncertainties and Errors in the TDOA due to Measuring Process

It should be noted that the measurement of the TDOA underlays a digital processing of the PD signal that includes sampling. Taking into account that partial discharges can have rise times well below hundreds of picoseconds, the signals have to be acquired with high-speed oscilloscopes or digitizers. Still, in this scenario, the sampling time may eventually prompt uncertainty: there will be a region in space given by the sampling time $T_{s}$ where we will be unable to determine the position of the source. The sampling frequency used in this paper is 5 GS/s, which corresponds to a sampling time of 200 ps. The differences in the TDOA below this time resolution will not be detected. Considering that the speed of propagation is the speed of light, *c*, the resolution in distance is 6 cm.

An additional error source in the detection of the signal onset may be present if the relation between the PD waveform bandwidth and the sampling frequency is not adequate. Digitalization implies missing information of the signal evolution between samples, which could be relevant if the sampling frequency is too low. As an example, we may be unable to pick the initial time if there is a significant change in the signal waveform between two consecutive samples.

It may be possible to mitigate artificially these shortcomings increasing the resolution with cubic splines interpolation [13,23], though the number of points introduced in the interpolation is limited.

Additionally, systematic errors in the digitizing process together with the uncertainty of the instrumentation, coaxial cable lengths and differences in the antennas properties can also modify the value of the TDOA.

## 3. Antenna Deployment and Sensitivity to Measurement Errors

Finding the position of the source using four antennas is a geometric problem. All possible solutions are placed at the intersection of hyperbolas with foci in every pair of antennas. The problem of finding the source is much harder when there are uncertainties in the determination of the TDOA. Then, depending on the relative positions of the antennas, there would be certain areas where the concentration of possible solutions would be denser (resulting in lower errors in the distance to the source) and regions where the concentration would be sparser (resulting in larger errors in the distance) [13]. Then, to have an accurate localization, the array of the antennas should be oriented to the directions in which the uncertainties are lower.

In this section we will analyze different antenna deployments and study the performance of each configuration in terms of accuracy and dispersion of the position of the source by simulation. We assume that there is a strong line-of-sight and that reflections are attenuated and delayed in such a way that they do not interfere in the free-space assumption. Also, we work under the premise that all other errors and uncertainty sources described in the last Section will be encompassed in a single error parameter given by the sampling time, $T_{s}$, of the PD signal. Then, the target of this Section is to evaluate what antenna array is best under the same conditions independently of the nature of the error. Under these circumstances, the results will show that choosing an appropriate layout can reduce the uncertainty in the location of the source due to errors in the TDOA.

Finding analytically the sensitivity to the different error sources in the TDOA in Equation (3) to obtain all possible deviations in the source position is a difficult task. Moreover, although the source can be located with only three equations, there is a total of six TDOA that can be involved in the solution of the non-linear system. The proposed approach assumes an error of one time sample, $|\epsilon_{ij}|=T_{s}$, sequentially in all six TDOA. Then, there would three possible states for every TDOA: $t_{i}-t_{j}-\epsilon_{ij}$, $t_{i}-t_{j}$, and $t_{i}-t_{j}+\epsilon_{ij}$ giving a total of 3^6^ = 729 possible solutions. All possible errors in the range $\pm \epsilon_{ij}$ are not repeated and they explore all the locations of the source. A common assumption when dealing with noise and errors is to assume that smaller errors are the most likely, a straightforward example of that is the Gaussian distribution; thus, the simulation is done with the minimum possible error, $|\epsilon_{ij}|=T_{s}$, since larger errors would give a cluster of possible positions too vast and set far from the actual location losing its practical applicability. Therefore, Equation (2) would be changed to $D_{ij}^{\prime }-∥P_{i}-P_{s}^{\prime }∥+∥P_{j}-P_{s}^{\prime }∥=0$ where $D_{ij}^{\prime }$ is the distance including the error $\pm \epsilon_{ij}$ and $P_{s}^{\prime }$ is the position of the source with the error in the TDOA. Let ${\hat{P}}_{s}=\left({\hat{x}}_{s},{\hat{y}}_{s},{\hat{z}}_{s}\right)$ be the estimation of its position, then setting the same equation for all pairs of antennas and summing all equations together gives:

(4) $$f\left({\hat{x}}_{s},{\hat{y}}_{s},{\hat{z}}_{s}\right)=\sum_{i=1}^{L-1}\sum_{j=i+1}^{L}{\left(D_{ij}^{\prime }-∥P_{i}-{\hat{P}}_{s}∥+∥P_{j}-{\hat{P}}_{s}∥\right)}^{2}$$

where *L* is the number of antennas and the distance differences have been squared to consider only positive values in the objective function. Equation (4) would be 0 for the correct estimation of ${\hat{P}}_{s}$, so any method that minimizes $f({\hat{x}}_{s},{\hat{y}}_{s},{\hat{z}}_{s})$ would give the estimated position of the source. Particle swarm optimization (PSO) is a feasible option to obtain good results as shown in [23].

**Figure 1:**
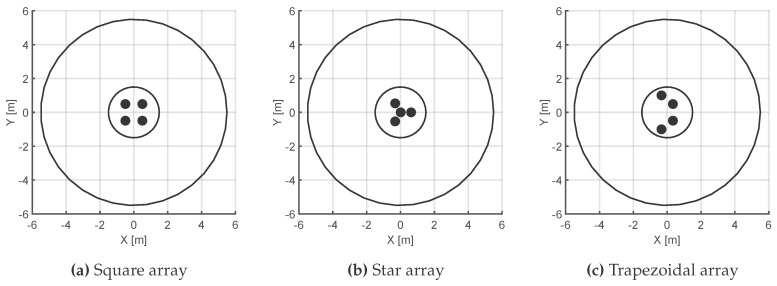
Three scenarios to test the performance of the antenna arrays. The radii of the circumferences are 1.5 m and 5.5 m.

The behavior of three different antenna deployments were tested for positions of the source on the circumferences around the antennas, Figure 1. All formations are balanced so none of them is favoured in terms of their relative position to the source. This is ensured by keeping the area of the array constant to one square meter. In the case of the star arrangement, there is an antenna in the center that has also to be considered, so the total area is the area of the external equilateral triangle plus the areas of the three isosceles triangles formed by the perimetrical antennas and the central one.

In all scenarios the PSO algorithm is run every 10° along the circumferences to obtain all possible solutions when there is an error of $\pm \epsilon_{ij}$ in the TDOA. Then, the solutions are analyzed statistically calculating the distance of the mean value in each of the coordinates to the actual position of the source as a measure of the uncertainty in the localization; and the mean value of the distances of all possible solutions to the actual position, as a measure of the dispersion of the data. These parameters would determine whether there is a configuration that stands out from others in finding the source with higher accuracy and less dispersion due to errors in the measurement of the TDOA. All antennas are in the same plane so the component *z* is not relevant in this study and the paper focuses the results on the plane *XY*. To have an adequate resolution in the *Z*-axis, at least one of the antennas should be placed in a different plane.

**Figure 2:**
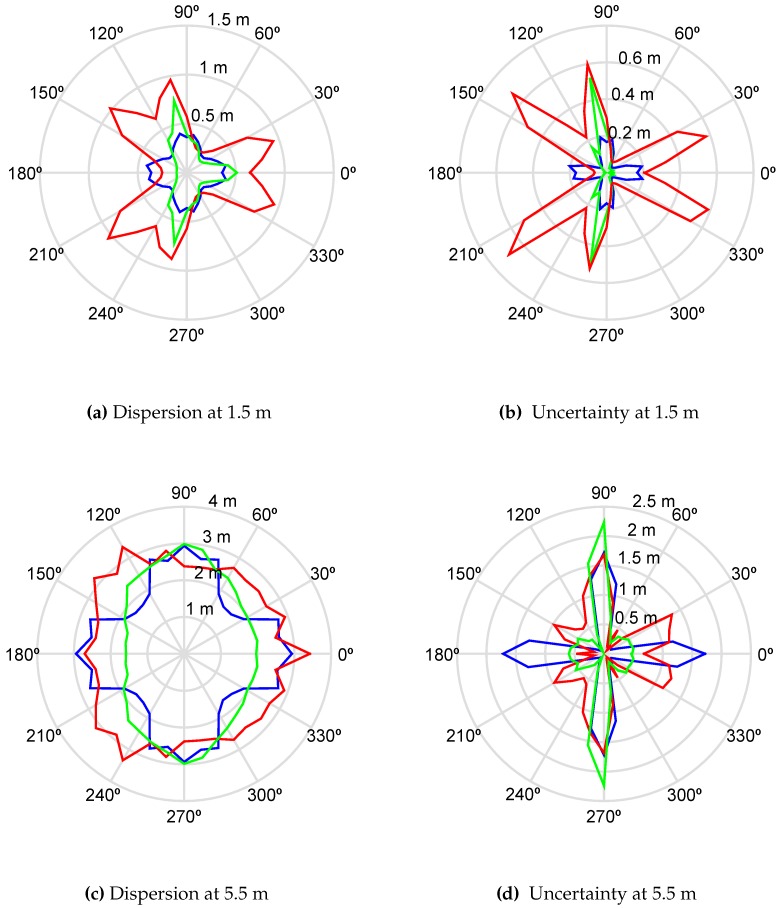
Simulation results for the three antenna layouts and the source located in circumferences with radii of 1.5 and 5.5 cm. The first column are the average dispersion of the solutions and the second column are the uncertainties of the average of the solutions. The layout in square is represented in blue, the star in red and the trapezoid in green.

Figure 2 shows the simulation results for dispersion and uncertainty measured in meters of all possible positions of the source when it is actually placed at 1.5 m and 5.5 m from the center of the layout. These polar plots are very helpful to find the directions in which the antennas have the best performance. The red line corresponds to the star formation. The preferable relative positions of the source in terms of dispersion of the results are 60°, 180° and 300°, plot Figure 2a. This means that, when the TDOA have errors of $\pm \epsilon_{ij}$ samples, all possible positions of the source are less scattered in those angles. Additionally, plot Figure 2b shows the uncertainty as the mean of all possible positions with respect to the actual position of the source. The best performance of the star formation is again in the same directions as in the dispersion plot plus 0°, 120° and 240°. When the distance is increased to 5.5 m, the measure of the scatter tends to be uniform in all directions, plot Figure 2c.

The blue line corresponds to the square array. In general, the performance of this formation is much better than the star’s as long as the directions around 0°, 90°, 180° and 270° are avoided. This would mean that if the partial discharge source is bearing for those angles, the square array would have problems in finding its position accurately and the possible positions would be scattered along those directions. Notice that the accuracy in finding the position of the source is very sharp between 15° and 75° and repeated in the rest of the quadrants, Figure 2b. When the source is located at 5.5 m, the arc of sharp accuracy is slightly extended, plot Figure 2d.

Finally, the green plot corresponds to the trapezoidal arrangement. The scattering with the source at 1.5 m is similar to the formation in square in quadrants 1 and 4 and the performance is better from 120° to 240° in the second and third quadrants. There are two narrow circular sectors, though, where the square beats the trapezoid, [90°, 120°] and [240°, 270°]. For longer distances, the behavior of the trapezoidal array is noticeable better when the parallel sides of the formation are perpendicular to the source position. However, the advantages of the trapezoid are better appreciated in the accuracy of the detection of the source. The differences between the actual position of the source and the average of the possible positions in the simulation is lower than 10 % (15 cm or 55 cm depending on the distance) for almost any bearing. Only for a narrow sector in [90°, 100°] and [260°, 270°], are the deviations important and similar to those of the star configuration, though still better than the squared one. A similar behaviour is observed when the source is located 5.5 m away.

In summary, considering that in a substation the source position in unknown, the initial orientation of the array of antennas would be random and it would be impossible to know if the source is in a favourable direction. Under those circumstances, the results in Table 1 show that the trapezoidal array has the overall best average accuracy and dispersion of the three configurations and distances. Moreover, the behavior of this configuration in the broad sectors [−80°, 80°] and [110°, 250°] is notably better than the other two. Consequently, the low uncertainty and high accuracy of the trapezoidal array in that sectors, totalling 300°, would permit a better localization of the source.

## 4. Experimental Study

**Figure 3:**
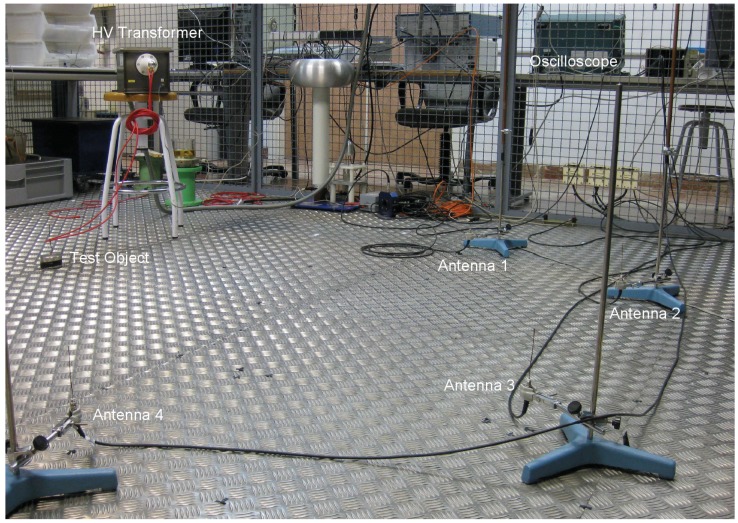
Antennal layout in the LINEALT high-voltage laboratory.

The three antenna array layouts were tested in a laboratory, Figure 3. The antennas were monopoles 10 cm long which are omnidirectional and have good response in the range of frequencies where partial discharges emit [24]. All coaxial cables have the same length and are connected to an oscilloscope with four channels.

The partial discharge source is created using a 25 kV high-voltage cable connected to a voltage-controlled transformer. A copper wire is bent to form a ring around the high-voltage cable, and then connected to ground. The resulting test object will have a high electric field divergence in that loop that will be able to create surface partial discharges [23].

The sampling frequency used in this paper is 5 GS/s, which corresponds to a sampling time $T_{s}=\left|\epsilon_{ij}\right|=200$ ps. Differences in the TDOA below this time resolution will not be detected. Considering that the speed of propagation is the speed of light, *c*, the resolution in distance is 6 cm. Interpolation can help to artificially increase the resolution and improve the results [13,23], so the sampling time is increased tenfold using cubic splines.

All measurements consist of a set of 500 partial discharges. One of the key aspects to have an accurate TDOA is the choosing of an automatic picker to determine the onsets of the pulses and, with them, the time differences. In this paper, the TDOA are calculated using the cumulative energy method with negative trend or Hinkley criterion that has been proved to give accurate results [23]. The TDOA are calculated for all the acquired signals to give a total of 500 sets of six TDOA. The location of the source is now estimated with the mode or the most repeated set of TDOA. It should be noted that we used the average position of all solutions in the simulation results for two reasons: any set of six TDOA can happen only once so the mode would not exist and the actual position of the source is known, so it is possible to calculate the exact deviation of all solutions with respect to the correct one through the average. Now, there are certain sets of TDOA that never happen due to imperfections in the antennas, the metallic holders that support the antennas and the metallic surfaces that surround the setup. This is translated into a distribution of frequencies of occurrence of the sets of TDOA that is not uniform as in the case of the simulation. Consequently, the mode is preferred to the average in a real experiment. As done in the simulation, all signals having a difference greater than $\pm \epsilon_{ij}$ that referred to the mode are discarded. This is done to emulate the results presented in the previous section. The remaining sets of TDOA are used to locate the source minimizing Equation (4) using PSO.

**Figure 4:**
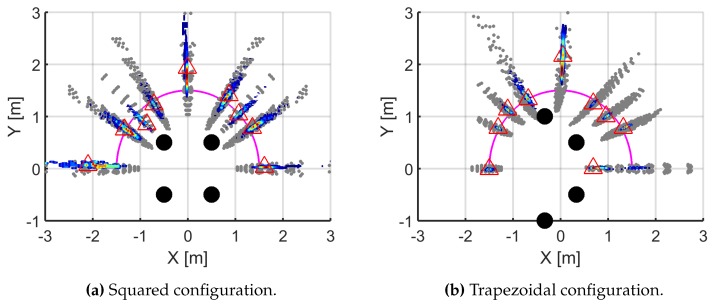
Simulated and experimental results for the squared and trapezoidal deployments.

Considering that the star array had a poor simulated performance, the experimental measurements were taken only for the formations in square and trapezoid. The partial discharges source was placed in selected points on a semi-circumference around the antennas and the radius was set to 1.5 m due to room constraints in the laboratory. The results are shown in Figure 4 where the magenta line represents the positions of the source. The grey points are the outcome of the simulations when the TDOAs have errors of one sample; the points with colors ranging from dark blue to dark red correspond to the solutions given by the PSO for the TDOAs calculated from actual pulses. Dark blue points means that the concentration of solutions is low whereas dark red ones means that there are many solutions in that area so the source would very likely be there. The triangles mark the mode of all experimental solutions which would be where the source is located based on those measurements.

Some conclusions can be drawn from Figure 4. First, all points obtained experimentally fall inside the cluster defined by the analysis done in Section 3 though they do not cover all possible solutions of the simulation. As explained before, this means that the deviations of the TDOA in the experimental measurements are not uniformly distributed and there are sets of TDOA that are repeated more than others. This also has the consequence that, in the 90° direction in the case of the trapezoidal configuration where the dispersion is large, the set of solutions do not intersect with the actual position in the magenta line. Second, the size of the clusters of the experimental outcome is noticeably smaller in the trapezoid than in the square in all directions except in the interval [90°, 120°] as expected from the simulations. This favors the use of this type of configuration because the position of the source is found with less uncertainty. Third, the accuracy of the trapezoidal formation given by the position triangles in the Figure is again better than in the square except in 0° and 90°. This was expected in the latter direction, 90°, as predicted by the simulations but in the bearing for 0° the trapezoid should have placed the source in its exact point as suggested the green plot in Figure 2b. This can be explained examining in detail the possible solutions for this bearing: there are two clusters, one of them has more probability of occurring so the mode (triangle) is placed on it. The other cluster in 0°, in blue colors, contains the correct set of TDOA of the real position of the source but, unfortunately, the PSO algorithm favors the wrong location.

## 5. Conclusions

Choosing an adequate layout of antennas can help in the localization of PD sources. The results highlight that the dispersion and specially the accuracy in the estimations of the locations of the PD emitter show an strong dependence on the relative position of the source which respect to the antenna array. Consequently, each configuration has its own optimal orientation for the localization of events. This paper also shows that the trapezoidal configuration can reduce the dispersion of the possible solutions of the source better than other configurations such as a square or a star. Additionally, the average value of all data in the components *x* and *y* is also closer to the actual position of the source in most of the directions considered. If the partial discharge source is far from the antenna array, the performance of the trapezoidal configuration is also better, which is very appropriate when measuring this type of events in open air substations. The study in the axis *Z* has been omitted because all antennas are in the same plane; at least one of the antennas has to be placed outside the horizontal plane to have sufficient resolution in height if localization in space is intended. The proposed method helps to define the best deployment of antennas in the maintenance of assets in open-air substations with the aim of finding which piece of equipment has partial discharges. Once it has been localized, an alternative method based on the classical setups defined in IEC 60270 [3] could be used to make a more accurate diagnosis towards the identification of the type of PD. In the specific case of transformers, where the discharges usually occur inside the tank, the antennas would register the external radiation of the PD when they are conducted through the windings to the bushings or the external connections. A more accurate localization of the source could be done with another array of antennas placed inside the tank.

## Figures and Tables

**Table 1:** Average performance in all directions and two broad circular sectors for the three configurations of antennas. All data are shown in meters.

	Dispersion 1.5 m			Uncertainty 1.5 m			Dispersion 5.5 m			Uncertainty 5.5 m		
	Star	Sq.	Trap.	Star	Sq.	Trap.	Star	Sq.	Trap.	Star	Sq.	Trap.
[−80°, 80°]	0.50	0.31	0.28	0.23	0.10	0.05	2.66	2.26	2.23	0.69	0.52	0.43
[110°, 250°]	0.61	0.30	0.17	0.30	0.09	0.04	2.83	2.15	1.97	0.60	0.44	0.39
Overall	0.59	0.32	0.28	0.29	0.11	0.09	2.71	2.30	2.22	0.77	0.59	0.57
